# Tumor shrinkage after simultaneous proton therapy for multiple hepatocellular carcinomas

**DOI:** 10.1093/jrr/rraf044

**Published:** 2025-07-23

**Authors:** Hikaru Niitsu, Masashi Mizumoto, Yinuo Li, Taisuke Sumiya, Keiichiro Baba, Motohiro Murakami, Masatoshi Nakamura, Toshiki Ishida, Takashi Iizumi, Takashi Saito, Haruko Numajiri, Hirokazu Makishima, Kei Nakai, Yoshiko Oshiro, Hideyuki Sakurai

**Affiliations:** Proton Medical Research Center, Department of Radiation Oncology, University of Tsukuba Hospital, Tsukuba 305-8576, Ibaraki, Japan; Proton Medical Research Center, Department of Radiation Oncology, University of Tsukuba Hospital, Tsukuba 305-8576, Ibaraki, Japan; Proton Medical Research Center, Department of Radiation Oncology, University of Tsukuba Hospital, Tsukuba 305-8576, Ibaraki, Japan; Proton Medical Research Center, Department of Radiation Oncology, University of Tsukuba Hospital, Tsukuba 305-8576, Ibaraki, Japan; Proton Medical Research Center, Department of Radiation Oncology, University of Tsukuba Hospital, Tsukuba 305-8576, Ibaraki, Japan; Proton Medical Research Center, Department of Radiation Oncology, University of Tsukuba Hospital, Tsukuba 305-8576, Ibaraki, Japan; Proton Medical Research Center, Department of Radiation Oncology, University of Tsukuba Hospital, Tsukuba 305-8576, Ibaraki, Japan; Proton Medical Research Center, Department of Radiation Oncology, University of Tsukuba Hospital, Tsukuba 305-8576, Ibaraki, Japan; Proton Medical Research Center, Department of Radiation Oncology, University of Tsukuba Hospital, Tsukuba 305-8576, Ibaraki, Japan; Proton Medical Research Center, Department of Radiation Oncology, University of Tsukuba Hospital, Tsukuba 305-8576, Ibaraki, Japan; Proton Medical Research Center, Department of Radiation Oncology, University of Tsukuba Hospital, Tsukuba 305-8576, Ibaraki, Japan; Proton Medical Research Center, Department of Radiation Oncology, University of Tsukuba Hospital, Tsukuba 305-8576, Ibaraki, Japan; Proton Medical Research Center, Department of Radiation Oncology, University of Tsukuba Hospital, Tsukuba 305-8576, Ibaraki, Japan; Proton Medical Research Center, Department of Radiation Oncology, University of Tsukuba Hospital, Tsukuba 305-8576, Ibaraki, Japan; Department of Radiation Oncology, Tsukuba Medical Center Hospital, Tsukuba 305-8558, Ibaraki, Japan; Proton Medical Research Center, Department of Radiation Oncology, University of Tsukuba Hospital, Tsukuba 305-8576, Ibaraki, Japan

**Keywords:** proton beam therapy, multiple hepatocarcinomas, tumor shrinkage, radiation dose

## Abstract

There are no reports on shrinkage of multiple hepatocellular carcinomas (HCCs) after simultaneous treatment with radiotherapy. The purpose of the study was to examine the relationship between tumor shrinkage and treatment outcomes for several HCCs irradiated simultaneously using proton beam therapy (PBT). The subjects were 46 patients with multiple HCCs (95 lesions) who received PBT between January 2008 and December 2018. Overall survival (OS), local control (LC) and complete+partial response (CR + PR) rates were determined using the Kaplan–Meier method. The median follow-up period was 29.2 months and the 3-year OS was 50.3%. For the 95 lesions, the 3-year LC rate was 90.4% and the CR + PR rate was 85.2% at 3 years. Three combination protocols (referred to as A, B and C) were used for different lesions in the same patient: A (66 Gray (Gy) Relative biological effectiveness (RBE) in 10 fractions (fr))-B (72.6 Gy(RBE) in 22 fr) (22 lesions), A-C (74 Gy(RBE) in 37 fr) (15 lesions) and B-C (2 lesions). The 1-year CR + PR rates were 75.8% for A and 56.4% for B in A-B cases (*P* = 0.14), and 62.5% for A and 57.1% for C in A-C cases (*P* = 0.35). In the B-C group, there was only one patient with 2 lesions. The lesion treated with the B protocol reached CR + PR, while that treated with the C protocol did not reach CR + PR. These results show that some cases can have differences in tumor shrinkage after concurrent PBT for multiple HCCs, and that there is no significant relationship between dose and tumor shrinkage.

## INTRODUCTION

Primary liver cancer is the third most common cause of cancer death worldwide, and hepatocellular carcinoma (HCC) accounts for ⁓80% of primary liver cancer [1]. Radiofrequency ablation and resection are the standard locoregional treatments for HCC [2–4]. Radiotherapy is an option in cases that are difficult to treat using these methods, and the efficacy and safety of particle therapy has been shown [5–7]. However, tumor shrinkage is generally slow, and enhancement on arterial phase at contrast-enhanced computed tomography (CT) or magnetic resonance imaging (MRI) remains after radiotherapy for HCC. There are no specific methods and timing for evaluation after radiotherapy.

The relationship between outcomes and tumor shrinkage has been examined in some reports [8, 9]. In the past report of this relationship after proton beam therapy (PBT), single tumors or larger lesions in cases with multiple lesions were analyzed. In some cases, the tumor shrinkage may continue after 1 year. Thus, it is important to continue follow up with CT and MRI beyond 1 year after PBT [9].

For multiple HCCs, there is a need to consider dose overlap in the liver. The efficacy and safety of radiation for multiple HCCs and repeated irradiation have been examined [10, 11], and the rate of shrinkage often differs for each lesion. If the tumor diameter and dose fractionation are similar, there is no difference in tumor shrinkage, but there may be lower tumor shrinkage with a larger tumor diameter and higher tumor shrinkage with a higher dose per one fraction. Differences in tumor shrinkage in simultaneous radiotherapy for multiple HCCs have not been examined. Therefore, in this study, we examined the relationship of tumor shrinkage with treatment outcomes for several HCCs after simultaneous irradiation.

## MATERIALS AND METHODS

The subjects were patients with multiple HCCs who were treated with PBT between January 2008 and December 2018 at our hospital. All patients underwent CT/MRI before and after PBT, and all images were available for evaluation in our medical records system. Post-treatment evaluations at our hospital were made by radiologists, and evaluations at other hospitals were made by radiation oncologists.

For treatment planning, follow-up and analysis, we used the same method as that in the past report [9]. The following three protocols were used: protocol A, 66 Gray (Gy) Relative Biological Effectiveness (RBE) in 10 fractions (fr) for peripheral lesions located distal to the gastrointestinal tract and porta hepatis; protocol B, 72.6 Gy (RBE) in 22 fr for hilar lesions; and protocol C, 74 Gy (RBE) in 37 fr for lesions requiring avoidance of gastrointestinal damage. CT, MRI and blood tests were performed every 3 months for the first 2 years after PBT and every 6 months thereafter.

This study included cases with multiple lesions that were irradiated simultaneously. The lesions were evaluated using the Response Evaluation Criteria in Solid Tumors (RECIST) criteria by measuring the largest diameter. Overall survival (OS) and local control (LC) rates were determined using the Kaplan–Meier method. Local recurrence was defined as tumor enlargement or early enhancement on imaging. The complete+partial respnse (CR + PR rate is ≥30% reduction of tumor size), 100% reduction (CR) rate and 50% reduction rate, including patients lost to follow-up and those who died before CR + PR or 100% or 50% reduction, were calculated with the Kaplan–Meier method. A Fine-Gray regression model was used to estimate the hazard ratio and 95% confidence interval (CI). A χ-square test for examination of the relationship between the concordance in protocol and concordance in tumor reduction.

## RESULTS

A total of 755 patients with 894 HCC lesions were treated with PBT at our center from January 2008 to December 2018. This study included 46 patients with 95 lesions treated with concurrent PBT for multiple lesions, including 44 patients with 88 lesions who received concurrent PBT for two lesions, one patient with three lesions who received concurrent PBT for triple lesions and one patient with four lesions who received concurrent PBT for two lesions at two times. The characteristics of these cases are summarized in Table 1. In terms of recurrence patterns, there were two cases of local recurrence, three cases of local recurrence and intrahepatic recurrence, 32 cases of intrahepatic recurrence and nine cases of no recurrence. In two cases of local recurrence, one case received no treatment and one case underwent TACE. In three cases with local recurrence and intrahepatic recurrence, one case received re-irradiation alone, one case received re-irradiation followed by molecular targeted agent (MTA) and one case received radiofrequency ablation (RFA) followed by trans arterial chemoembolization (TACE). Among the 32 cases of intrahepatic recurrence, seven cases received no treatment, 17 cases underwent locoregional treatment (hepatectomy, RFA, TACE, PBT), seven cases received locoregional treatment followed by systemic therapy and one received systemic therapy alone. In the nine cases received systemic therapy, five cases received sorafenib, two cases received MTA (lenvatinib and sorafenib) and immune checkpoint inhibitors (the combination of atezolizumab and bevacizumab), and two cases received chemotherapy (the combination of fluorouracil and cisplatin). The median follow-up period was 29.2 months (range: 4.4–126.9 months). OS was 93.3% (95% CI: 89.5–97.1%) at 1 year, 74.2% (67.5–80.9%) at 2 years and 50.3% (42.3–58.3%) at 3 years.

**Table 1:** Characteristics of 46 patients with 95 lesions

Characteristics	Number	%
Age (years)	21–89	73 (median)
Gender		
Male	36	78.3
Female	10	21.7
ECOG performance status		
0	28	60.9
1	15	32.6
2	3	6.5
History of hepatitis		
Non-B, non-C	14	30.4
HCV	26	56.5
HBV	5	13.0
Child–Pugh class		
A	34	73.9
B	12	26.0
Tumor location		
Peripheral	27	28.4
Hepatic portal	54	56.8
Gastrointestinal proximity	14	14.8
Tumor size (mm)	6–85	23 (median)
Portal vein tumor thrombus		
Vp 0–2	40	87.0
Vp 3–4	6	13.0
Surgical indication		
Operable	6	13.0
Inoperable	40	87.0
Prior treatment		
Yes	23	50.0
No	23	50.0
Prior radiotherapy		
Yes	8	17.4
No	38	82.6
Clinical stage		
II	31	67.4
III	13	28.3
IV	2	4.3

ECOG, Eastern Cooperative Oncology Group; HCV, hepatitis C virus; HBV, hepatitis B virus.

For the 95 lesions, the 1-, 2- and 3-year LC rates were 98.8%, 96.0% and 90.4%. Regarding the relationship between LC and tumor reduction, the 3-year LC rates were 100% vs. 88.3% in CR vs. non-CR cases at 6 months (*P* = 0.236), 93.9% vs. 87.5% in PR vs. non-PR cases at 6 months (*P* = 0.543), 100% vs. 86.0% in CR vs. non-CR cases at 12 months (*P* = 0.089) and 95.3% vs. 83.6% in PR vs. non-PR cases at 12 months (*P* = 0.195). There was no significant difference for any of these comparisons.

**Fig. 1:**
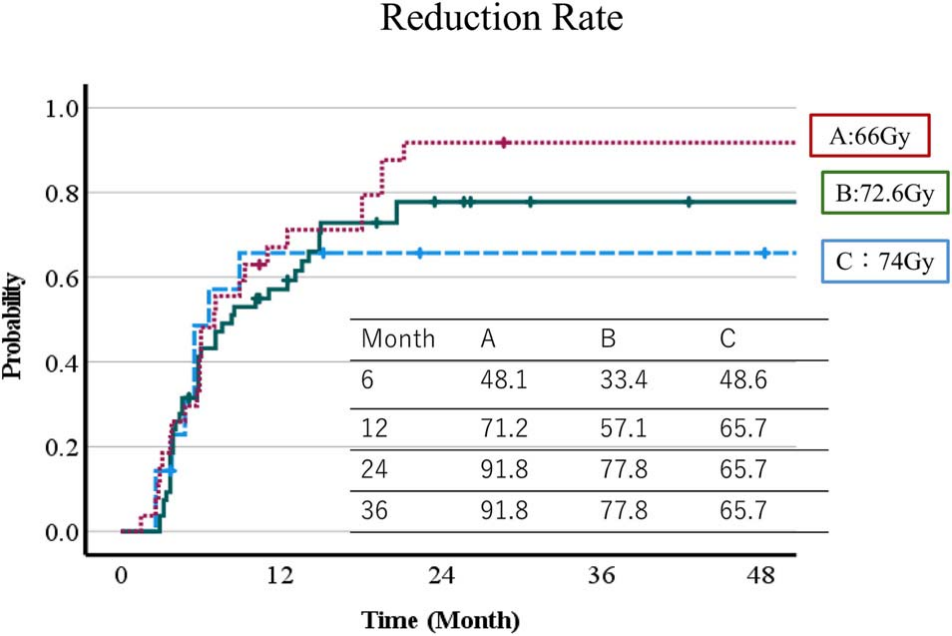
Complete+partial response (CR + PR) rate by protocol.

The CR + PR rates were 33.1% at 6 months, 57.5% at 1 year, 76.9% at 2 years and 85.2% at 3 years. The respective CR + PR rates were 48.1%, 71.2%, 91.8% and 91.8% with protocol A (66 Gy (RBE) in 10 fr); 33.4%, 57.1%, 77.8% and 77.8% with protocol B (72.6 Gy (RBE) in 22 fr); and 48.6%, 65.7%, 65.7% and 65.7% with protocol C (74 Gy (RBE) in 37 fr). There were no significant differences among the three protocols (*P* = 0.507). The CR + PR rate of each protocol is shown in Fig. 1.

**Fig. 2:**
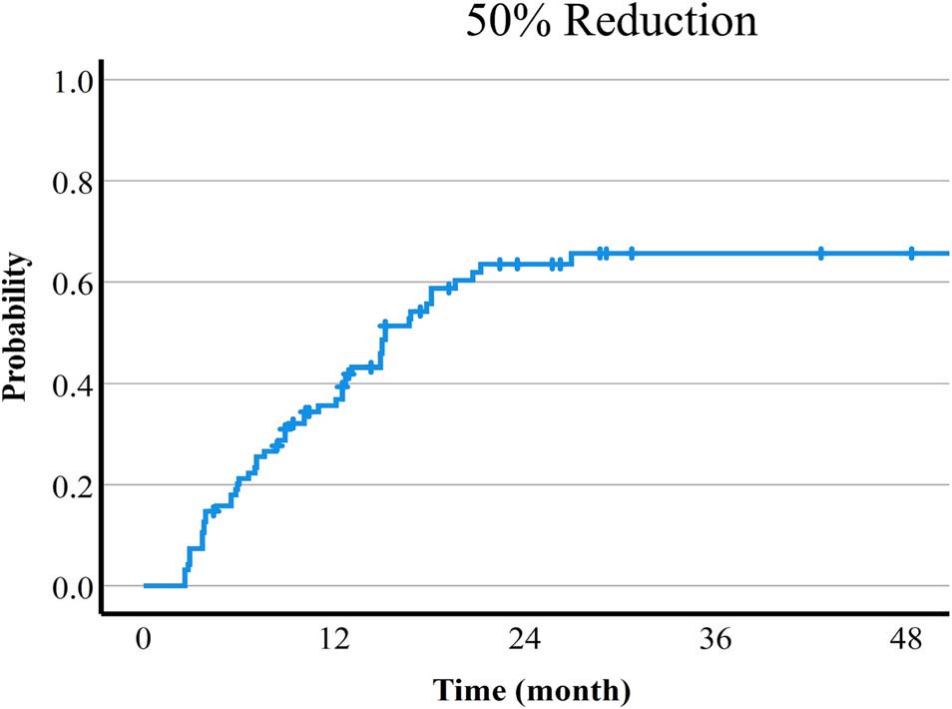
Time to 50% reduction of the long diameter of the tumor.

**Fig. 3:**
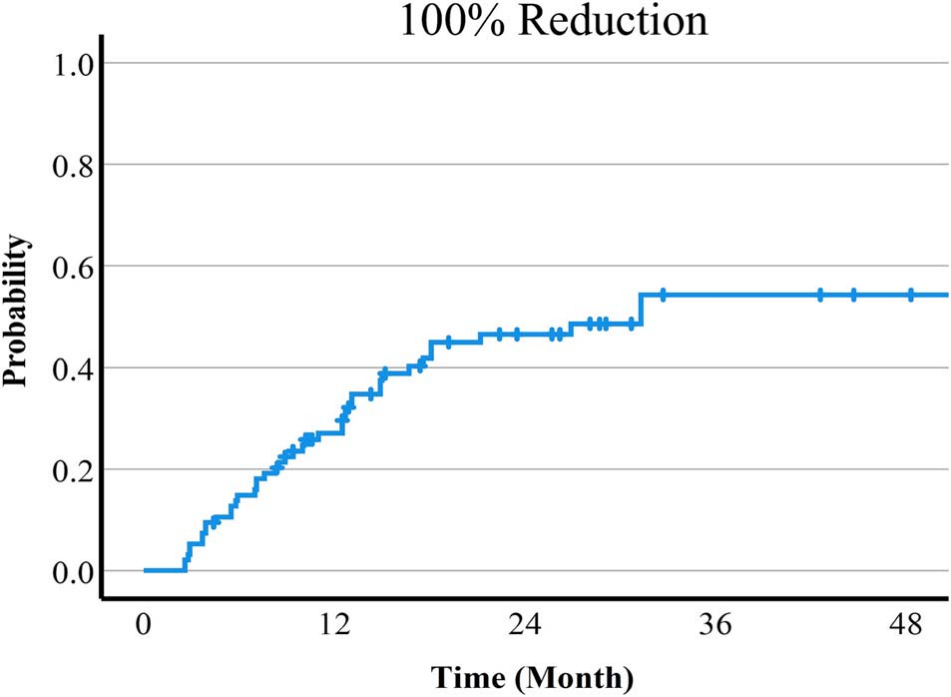
Time to 100% reduction of the long diameter of the tumor.

Times to 100% or 50% reduction of the long diameter of the tumor are shown in Figs. 2 and 3. The 1-, 2- and, 3-year 100% and 50% reduction rates were 27.1%, 46.5% and 54.2%, and 35.6%, 63.5% and 65.6%, respectively. The pre-treatment tumor size was significantly associated with the time to 50% or 100% reduction. Furthermore, there was the trend that the 66Gy protocol could achieve 50% reduction more easily than the 74Gy protocol, but there was no significant difference. There was no relationship between systemic therapy and tumor reduction (Table 2). A list of cases with differences in tumor shrinkage in the same patient is shown in Table 3. There was a trend for better shrinkage for tumors with smaller diameters and those that received the 66 Gy protocol.

**Table 2:** Fine–Gray regression model for factors associated with time to 50% or 100% reduction in the long diameter of the tumor

Factors	HR	*P*-value
50% Reduction		
Protocol A (control C)	2.54 (0.95–6.80)	0.063
Protocol B (control C)	1.05 (0.57–1.91)	0.88
Tumor size	0.64 (0.51–0.79)	<0.01
Systemic therapy	1.19 (0.62–2.29)	0.61
100% Reduction		
Protocol A (control C)	2.13 (0.56–8.09)	0.27
Protocol B (control C)	0.92 (0.48–1.74)	0.79
Tumor size	0.41 (0.29–0.58)	<0.01
Systemic therapy	0.80 (0.36–1.76)	0.57

Protocol A: 66 Gray (Gy) relative biological effectiveness (RBE)/10 fractions (fr), B: 72.6 Gy (RBE)/22 fr, C: 74 Gy (RBE)/37 fr.

**Table 3:** Cases with inconsistent reduction

No	Dose	Size(cm)	RECIST	No	Dose	Size(cm)	RECIST
1	A	3.1	SD	11	A	2.9	CR
	B	5	PR		B	3.2	SD
2	B	8.5	SD	12	C	4.3	PR
	B	7.7	PR		A	1.8	CR
3	B	1.3	SD	13	B	2.5	SD
	B	1.5	PR		B	3.1	PR
4	A	2.3	CR	14	B	4.1	SD
	C	6.8	PR		A	0.8	CR
5	A	1.5	PR	15	B	2.8	SD
	A	1.4	CR		B	0.6	PD
6	B	1.6	CR	14	B	4.1	SD
	B	2.2	SD		A	0.8	CR
7	C	3.9	SD	15	B	2.8	SD
	A	2.1	CR		B	0.6	PD
	A	3.3	PR	16	B	2.3	PR
					B	1.5	CR
8	B	3.3	SD	17	A	1.8	CR
	A	2	CR		A	0.7	PD
9	B	1.3	CR	18	C	5	SD
	C	2.5	SD		A	1.6	CR
10	B	3.5	PR	19	B	6.9	PR
	B	0.8	CR		B	3.6	SD

Dose: Protocol A (66 Gray (Gy) relative biological effectiveness (RBE)/10 fractions (fr)), B (72.6 Gy (RBE)/22 fr), C (74 Gy (RBE)/37 fr).

RECIST, Response Evaluation Criteria in Solid Tumors; CR, complete response; PR, partial response; SD, stable disease.

**Fig. 4:**
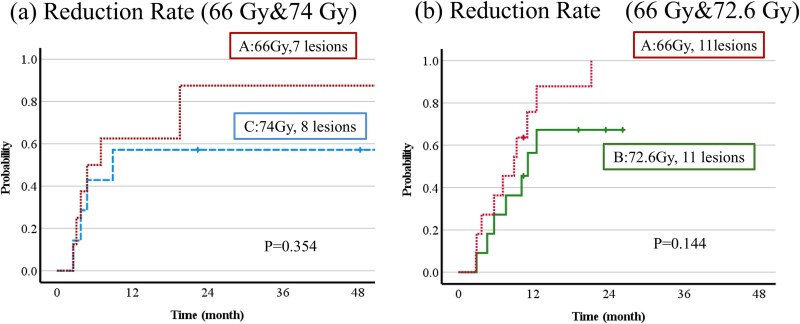
The combination of protocols used for each lesion in the same patients. (a) is protocol A and C, (b) is protocol A and B.

The combination of protocols used for each lesion in the same patient is shown in Table 4. For the different combinations, A-B was used for 22 lesions, A-C for 15 lesions and B-C for 2 lesions. The 1-year CR + PR rates in the different combinations were 75.8% for A and 56.4% for B in A-B cases (*P* = 0.14), and 62.5% for A and 57.1% for C in A-C cases (*P* = 0.35). The B-C group only had one patient with 2 lesions. The tumor treated with protocol B reached CR + PR while that treated with protocol C did not do so (Fig. 4). The relationship between the concordance in protocol and concordance in tumor reduction (Table 5) gave *P* = 0.070 and an association coefficient of φ = 0.186, which was not significant.

**Table 4:** Combination of protocols used for each lesion in the same patients

	A 66 Gy	B 72.6 Gy	C 74 Gy
A	8	22	15
B		42	2
C			6

**Table 5:** Relationship between concordance of protocol and concordance of tumor shrinkage

		Concordance of tumor shrinkage		
		Yes	No	Total
Concordance	Yes	44	12	56
of protocol	No	24	15	39
	68	27	95	

## DISCUSSION

This study focused on cases in which multiple lesions were simultaneously irradiated with PBT. The 1-, 2- and 3-year OS rates were 93.3%, 74.2% and 50.3%, respectively; and the. 1-, 2- and 3-year LC rates per lesion were 98.8%, 96.0% and 90.4%, respectively. These results are comparable to previous reports examining treatment of one HCC lesion [5–7, 9, 12, 13]. There are a few reports of simultaneous irradiation of multiple lesions of HCC [10, 14] and of irradiation at different times [11, 14–17]. Fukuda *et al.* found 5-year LC and OS rates for Barcelona Clinic Liver Cancer (BCLC) stage B (tumor length ≥ 3 cm, ≥4 lesions) of 87% and 66%, respectively [10]. In 11 patients with multiple lesions who received simultaneous irradiation, and 14 patients with irradiation at different times, Hall *et al*. found that 8 of 56 lesions recurred, and the median OS was 25 months [14]. Repeated PBT has been shown to give a 2-year OS of 87.5% [11], repeated stereotactic body radiotherapy (SBRT) gives a 3-year OS of 62.8% and a 3-year LC rate of 94.5% [15, 16], and carbon-ion radiotherapy has a 2-year OS of 87.8% and a 2-year LC rate of 83.0% [17]. There is a possibility of progression of HCC or decline of liver function in cases with repeated radiotherapy, and it is difficult to compare the previous results with those in the current study. We found no correlation between tumor reduction (CR or PR) at 6 or 12 months and treatment outcomes (OS or LC), and there was no relationship between the CR + PR rate and dose fractionation. However, there was the trend that the 66Gy protocol could achieve 50% reduction more easily than the 74Gy protocol, and 50% and 100% (CR) tumor reduction were correlated with tumor diameter. Based on these findings, the smaller tumors have higher radiosensitivity, and there is the possibility that tumor shrinkage is achieved at lower PBT dose. The larger tumors have required higher doses, but it could not be delivered sufficient doses, which could result in poor tumor shrinkage due to the risk of proximity to the portal vein and gastrointestinal tract. So, the correlation between PBT dose and tumor shrinkage could not be clarified. Previous studies of treatment of single tumors or larger lesions in cases with multiple lesions mentioned that 50% and 100% tumor reduction had an association with tumor diameter before PBT. And the reduction rate at 6 and 12 months was associated with OS, but there was no relationship with LC [9]. These results are similar to this study.

There are several reports of SBRT for multiple lesions in other organs [18–22], but none have examined differences in tumor shrinkage. In this study, there was a trend for higher CR + PR rates with protocol A (66 Gy (RBE)/10 fr) in cases irradiated with the A-B (72.6 Gy (RBE)/22 fr) and A-C (74 Gy (RBE)/37 fr) protocols, but with no significant difference. The B-C combination was only used for two lesions in one patient, and the lesion treated with protocol B had CR + PR, but the lesion that received protocol C had non-PR. A previous study mentioned that the respective CR + PR were 96.8% at 3 years with protocol A, 81.3% with protocol B and 82.0% at 3 years with protocol C. There was no relationship between tumor shrinkage and dose/fraction [9]. Thus, these results are similar to this study.

Tumor shrinkage did not show a significant correspondence to the dose/fraction, with some cases showing differences in tumor shrinkage using the same protocol in the same patients. There was a trend for more shrinkage with a higher dose per fraction, but there was no statistical correlation. The biological effectiveness (BED) is higher with a higher dose per fraction, and it is possible that protocols B and C give less tumor shrinkage, because reducing the dose to the hilar region and intestinal tract is likely to lower the dose to the tumor. Alternatively, the lack of a significant difference may be due to bias in selection of the protocols.

There are other limitations in the study, including its retrospective observational design and the possibility that factors other than irradiation is involved in tumor shrinkage. We considered a more comprehensive analysis of tumor shrinkage patterns stratified by tumor size, location and protocol in the future.

## CONCLUSION

Our results show that some cases have differences in tumor shrinkage after PBT for multiple HCCs in the same patient, and that there is no significant relationship between dose and tumor shrinkage.

## PRESENTATION AT A CONFERENCE

The 60th Annual Meeting of the Japan Liver Cancer Association.

## CLINICAL TRIAL REGISTRATION NUMBER

R05-230.
